# Preoperative and early postoperative albumin thresholds, rather than ΔAlb, predict severe complications after colorectal cancer surgery: a multifactorial model and nomogram

**DOI:** 10.3389/fonc.2026.1864078

**Published:** 2026-07-13

**Authors:** Lu Wang, Rui-Rui Lyu, Yue Ding, Ze-Qing Gao, Li-Qin Deng

**Affiliations:** 1 Department of Anesthesiology and Perioperative Medicine, General Hospital of Ningxia Medical University, First Clinical Medical College, Ningxia Medical University, Ningxia, China; 2 Department of Radiology, General Hospital of Ningxia Medical University, Ningxia, China; 3 Department of Anesthesiology and Perioperative Medicine, General Hospital of Ningxia Medical University, Ningxia, China

**Keywords:** colorectal cancer surgery, multifactorial comprehensive model, nomogram, postoperative complications, serum albumin dynamics

## Abstract

**Background:**

Postoperative complications of colorectal cancer significantly impact prognosis and healthcare burdens. While the dynamic change from preoperative to the lowest albumin level on postoperative day 2 (ΔAlb) has been linked to complications, their predictive value remains controversial. This study aims to explore the relationship between ΔAlb and postoperative complications, and evaluates a multifactorial predictive model for severe complications.

**Methods:**

This retrospective study analyzed 932 patients undergoing colorectal cancer resection (August 2020–April 2022). Data included preoperative/postoperative albumin levels, demographics, surgical/anesthetic details, and 30-day complications. ΔAlb’s association with severe complications was assessed via logistic regression, and a nomogram was developed.

**Results:**

Severe complications (Clavien–Dindo III–V) occurred in 14.2% of patients. ΔAlb showed no significant association with severe complications in univariate (P = 0.185) or multivariate analysis and discriminated poorly on ROC analysis (AUC 0.536, 95% CI 0.479–0.593). In the final multivariable model using clinically dichotomised predictors, preoperative albumin <35 g/L (OR 2.05, 95% CI 1.29–3.25, P = 0.003) and POD2 nadir albumin <28.5 g/L (OR 1.55, 95% CI 1.01–2.38, P = 0.044) were independent risk factors. Other independent predictors were age >65 years (OR 2.14, P = 0.003), ASA ≥3 (OR 2.14, P = 0.001), preoperative NRS2002 ≥2 (OR 1.97, P = 0.028) and rocuronium dose ≥117.5 mg (OR 4.44, P<0.001). A nomogram integrating these factors showed good discrimination (apparent C-index 0.794; optimism-corrected 0.784 by 1000-sample bootstrapping), good calibration (Hosmer–Lemeshow P = 0.72) and net clinical benefit on decision-curve analysis.

**Conclusion:**

ΔAlb alone has limited predictive value, whereas absolute preoperative albumin <35 g/L and POD2 nadir albumin <28.5 g/L are independent predictors of severe complications. A multifactorial model integrating these absolute albumin thresholds with age, ASA score, preoperative NRS2002 and rocuronium dose improves prediction (optimism-corrected C-index 0.78) compared with individual factors (AUC <0.7). Because rocuronium dose may also reflect surgical complexity and perioperative management, these associations require prospective, multicentre validation.

## Introduction

Colorectal cancer ranks third in incidence and second in mortality worldwide (1). Currently, approximately 70%–80% of cases are primarily treated with surgery (2). Although advancements in surgical techniques and the widespread adoption of Enhanced Recovery After Surgery (ERAS) protocols have significantly shortened postoperative hospital stays in recent decades, the incidence of postoperative complications has not shown substantial improvement (3). Complications such as surgical site infections, intra-abdominal infections, pneumonia, anastomotic leakage, anastomotic bleeding, and intestinal obstruction severely impact postoperative recovery, increasing the burden on families and healthcare systems (4). At present, effective tools for predicting the risk of postoperative complications in colorectal cancer remain lacking, limiting the ability to provide early warnings and reduce their occurrence.

Previous studies have suggested that early measurement of inflammatory mediators in peritoneal/abdominal drainage fluid can help predict anastomotic leakage in colorectal cancer surgery patients (5). However, this method is not applicable for assessing overall postoperative complications. C-reactive protein (CRP) and interleukin-6 are nonspecific inflammatory biomarkers that have been used to predict overall postoperative complications in colorectal cancer patients (6–8). However, their specificity is relatively low, and their kinetic response is slow, leading to a temporal mismatch between biomarker detection and the need for timely complication warnings.

Most existing studies have focused on static indicators while overlooking postoperative dynamic changes. In recent years, an inexpensive and readily available parameter—preoperative serum albumin and its dynamic changes during the postoperative period—has gained attention as a predictor of complications following major abdominal surgery (9). Wierdak et al. found that the change from preoperative to the lowest albumin level on postoperative day 2 (ΔAlb) could serve as an early predictor of infectious complications following laparoscopic colorectal cancer surgery (10). Similarly, Wang et al. reported that an early decline in serum albumin levels following radical laparoscopic colorectal cancer surgery was indicative of severe complications (11). However, a systematic review highlighted significant heterogeneity in the predictive thresholds of ΔAlb across different studies (12). Consequently, there remains insufficient evidence to determine whether perioperative changes in serum albumin levels can reliably predict postoperative complications in colorectal cancer.

This study aims to examine the association between the change from preoperative to the lowest albumin level on postoperative day 2 and postoperative complications in colorectal cancer. Additionally, we evaluate the predictive value of a multifactorial model in assessing the risk of severe postoperative complications in colorectal cancer patients. Because perioperative hypoalbuminemia is itself an established risk marker, it remains unclear whether absolute albumin thresholds or the relative change (ΔAlb) are more informative, and whether albumin adds predictive value when combined with intraoperative variables. We therefore directly compared absolute preoperative and early postoperative albumin thresholds with ΔAlb, and integrated albumin with intraoperative rocuronium dose into a single, internally validated nomogram, to provide a practical tool for early risk stratification.

## Methods

### Patients

**Figure 1:**
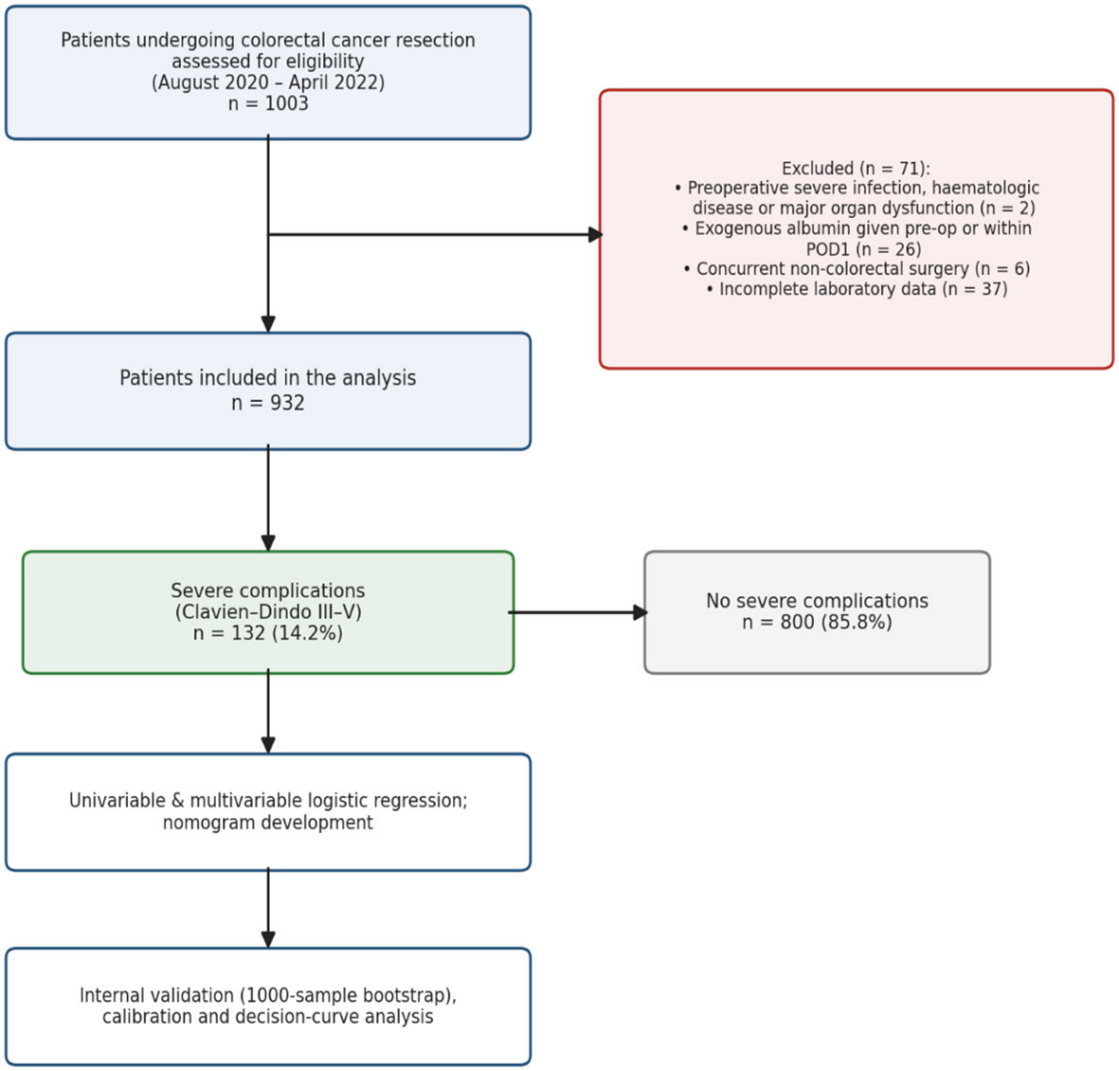
STROBE flow diagram of patient screening, exclusions and the final analytic cohort.

Patient screening, exclusions and the final analytic cohort are summarised in a STROBE-style flow diagram (**Figure 1**). This study protocol was approved by the Ethics Committee of the General Hospital of Ningxia Medical University (Approval No. KYLL-2021-1045); because the study was retrospective and used de-identified data, the requirement for written informed consent was waived by the Ethics Committee.

### Data collection

Surgical procedures were conducted according to the National Comprehensive Cancer Network (NCCN) Clinical Practice Guidelines, with intraoperative specimens collected for routine frozen section analysis (13). All enrolled patients received standardized perioperative care according to the Enhanced Recovery After Surgery (ERAS) protocol.

### Perioperative nutritional management

Preoperative nutritional assessment was performed using the Nutritional Risk Screening 2002 (NRS2002) at admission. Patients with preoperative NRS2002 score ≥ 2 or preoperative albumin <30 g/L were identified as high nutritional risk. For these high-risk patients, preoperative nutritional intervention was considered on an individualized basis, including oral nutritional supplements or enteral nutrition, at the discretion of the treating physician. However, no standardized preoperative nutritional supplementation protocol was mandatory during the study period.

Postoperatively, early oral intake was encouraged as per ERAS protocol, typically starting on postoperative day 1 (POD1) after the return of bowel sounds. Diet was resumed only after the patient passed flatus. Postoperative nutritional status was reassessed using the NRS2002 score within the first 48 hours. Patients with postoperative NRS2002 score ≥ 2 received enhanced nutritional support according to clinical judgment, which could include oral supplements, enteral nutrition, or parenteral nutrition as needed. The lack of a standardized postoperative nutritional protocol reflects real-world clinical practice and is acknowledged as a study limitation. No patients received exogenous albumin administration within POD2, as per exclusion criteria. The NRS2002 used in the regression model and the nomogram is the score obtained at the time of admission (preoperatively).

### Data variables

Demographic, laboratory, surgical, and anesthesia data were collected from each patient’s medical records. Demographic data included age, gender, body mass index (BMI), Charlson comorbidity index (CCI), hypertension, diabetes mellitus, American Society of Anesthesiologists (ASA) status, nutritional risk screening 2002 (NRS2002), chemotherapy history, and smoking history. Laboratory data included hematocrit, hemoglobin, neutrophil count, preoperative albumin, nadir albumin within postoperative day 2 (POD2), and albumin change. Surgical data included operation time, type of surgery, surgical approach, Tumor, Node, Metastasis stage (TNM), and stoma creation. Anesthesia data included anesthesia technique, anesthetic drugs, intraoperative blood transfusion, intraoperative fluid utilization, urinary output, and bleeding.

The calculation formula for albumin change (ΔAlb) was as follows: ΔALB = (preoperative Alb – lowest Alb within POD 2)/preoperative Alb × 100%.

### Definition of outcomes

Complications were recorded within 30 days postoperatively and graded according to the Clavien–Dindo classification; grade I–II events were considered minor complications and grade III–V events (including grade IV) were considered severe complications (14). Additionally, the Comprehensive Complication Index (CCI) was calculated for each patient (0–100) (15). Length of hospital stay was defined as the duration from admission to discharge. Surgical site infection (SSI) was defined according to the Centers for Disease Control and Prevention standards (16).

The primary research outcome is the relationship between ΔAlb and severe complications. Secondary research outcomes include the relationship between ΔAlb and overall complications, time to first bowel movement postoperatively, length of hospital stay, 30-day postoperative mortality rate, and SSI.

### Statistical analysis

Continuous variables were presented as mean ± SD or median (IQR) and compared using Student’s t-test or the Mann–Whitney U test; categorical variables were presented as frequencies (percentages) and compared using the chi-square or Fisher exact test. Candidate predictors were first screened by univariable logistic regression; variables with P<0.10 together with pre-specified clinically important factors were entered into a multivariable binary logistic regression, and the final model was confirmed by backward elimination. To match the nomogram and improve clinical usability, preoperative albumin, POD2 nadir albumin and rocuronium dose were dichotomised at ROC-derived cut-offs (maximum Youden index); the continuous-variable model was retained as a sensitivity analysis. Discrimination was assessed by the AUC and concordance index, and AUCs were compared using DeLong’s test. Model optimism was corrected by 1000-sample bootstrapping; calibration was evaluated using the calibration plot, calibration slope and Hosmer–Lemeshow test, and clinical utility by decision-curve analysis. Statistical significance was defined as P<0.05. All analyses were performed using SPSS 26.0 (SPSS, Chicago, IL, USA) and R 4.1.1 (https://www.r-project.org).

## Results

### Demographic and clinical characteristics

A total of 932 patients were included in the study, of which 132 (14.2%) experienced severe postoperative complications. Patients with severe complications were significantly older than those without severe complications (median age 72.0 vs. 65.0 years, P < 0.001). The proportion of patients with ASA ≥ 3 (70.5% vs. 42.9%, P < 0.001) and NRS2002 ≥ 2 (87.1% vs. 61.6%, P < 0.001) was significantly higher in the severe complication group. Furthermore, preoperative hemoglobin (Hgb) levels were lower in the severe complication group (125.1 vs. 130.0 g/L, P = 0.017), and preoperative albumin (36.4 vs. 38.3 g/L, P < 0.001) and nadir albumin within POD 2 (28.3 ± 4.3 vs. 30.3 ± 4.1 g/L, P < 0.001) levels were also significantly lower. A detailed breakdown of complication subtypes and the Comprehensive Complication Index by severity group is shown in **Table 1**.

**Table 1:** Complication subtypes and CCI by severity group.

Complication	Total n (%)	Severe (n=132)	Minor (n=800)	P value
Anastomotic leak	15 (1.6)	15 (11.4)	0 (0.0)	<0.001
Surgical-site infection	51 (5.5)	35 (26.5)	16 (2.0)	<0.001
Intra-abdominal infection	33 (3.5)	18 (13.6)	15 (1.9)	<0.001
Sepsis	6 (0.6)	6 (4.5)	0 (0.0)	<0.001
Pulmonary infection	47 (5.0)	35 (26.5)	12 (1.5)	<0.001
Hypoxaemia	30 (3.2)	26 (19.7)	4 (0.5)	<0.001
Respiratory failure	6 (0.6)	6 (4.5)	0 (0.0)	<0.001
Ileus	16 (1.7)	16 (12.1)	0 (0.0)	<0.001
Arrhythmia	14 (1.5)	10 (7.6)	4 (0.5)	<0.001
Hepatic insufficiency	7 (0.8)	7 (5.3)	0 (0.0)	<0.001
Renal injury	5 (0.5)	5 (3.8)	0 (0.0)	<0.001
30-day mortality	11 (1.2)	11 (8.3)	0 (0.0)	<0.001
CCI, median (IQR)	0 (0–8.7)	20.9 (8.7–33.5)	0 (0–0)	<0.001

Breakdown of postoperative complications and Comprehensive Complication Index (CCI) by severity group. Fisher exact test; patients may have more than one complication. Rare events (<0.5%) are summarised in the Supplement.

The types of surgeries performed also differed between the groups. A higher proportion of patients with severe complications underwent right colectomies (22.7% vs. 13.5%, P = 0.028), while laparoscopic approaches were less frequently employed in this cohort (82.6% vs. 89.6%, P = 0.025). Notably, patients who developed severe complications received higher intraoperative doses of rocuronium (median: 123.0 vs. 99.0 mg, P < 0.001) and experienced greater intraoperative blood loss (median: 144.0 vs. 121.6 mL, P = 0.035) (**Table 2**).

**Table 2:** Demographic, laboratory tests and intraoperative details for patients with severe complications.

Characteristic	Severe complications (n = 132)	No severe complications (n = 800)	P value
Demographic			
Age >65, y	95 (72.0)	326 (40.8)	<0.001
Men	79 (59.8)	477 (59.6)	0.961
BMI, kg/m^2^	23.5 ± 3.4	23.5 ± 3.2	0.973
CCI ≥3	12 (9.1)	42 (5.3)	0.080
Comorbidities			
Hypertension	51 (38.6)	252 (31.5)	0.105
Diabetes mellitus	23 (17.4)	126 (15.8)	0.805
ASA ≥3	93 (70.5)	342 (42.9)	<0.001
Preoperative NRS2002 ≥2	115 (87.1)	493 (61.6)	<0.001
Chemotherapy history	0 (0.0)	12 (1.5)	0.317
Smoking	21 (15.9)	138 (17.3)	0.704
Laboratory tests			
Preoperative Hct, %	38.4 (33.6-43.3)	40.0 (35.6-43.6)	0.062
Preoperative Hgb, g/L	125.5 (108.0-143.8)	132.0 (115.0-146.0)	<0.05
Preoperative Neu, ×10^9/L	3.4 (2.6-4.5)	3.4 (2.6-4.3)	0.715
Preoperative Alb, g/L	36.3 (33.7-39.5)	37.9 (35.6-41.0)	<0.001
Nadir Alb within POD 2, g/L	28.1 (25.3-31.5)	30.2 (27.6-33.0)	<0.001
△Alb	22.2 (12.8-31.5)	19.6 (12.4-27.6)	0.185
Operative details			
Operation time, median (range), min	220.0 (50.0-603.0)	219.0 (63.0-553.0)	0.630
Type of surgery			<0.05
Right colectomy	30 (22.7)	108 (13.5)	
Transverse colectomy	0 (0.0)	2 (0.3)	
Left colectomy	12 (9.1)	57 (7.1)	
Sigmoid resection	6 (4.5)	43 (5.4)	
Rectal resection	68 (51.5)	496 (62.0)	
Subtotal colectomy	1 (0.8)	0 (0.0)	
Abdominoperineal resection	9 (6.8)	72 (9.0)	
Other	6 (4.5)	22 (2.8)	
Laparoscopic surgery	109 (82.6)	717 (89.6)	<0.05
TNM stage			0.427
I-II	69 (52.3)	464 (58.1)	
III	52 (39.4)	282 (35.3)	
IV	11 (8.3)	53 (6.6)	
Stoma creation	33 (25.0)	269 (33.6)	0.056
Anesthesia details			
Anesthesia Technique			0.246
General anesthesia	122 (92.4)	699 (87.4)	
Local infiltration with general anesthesia	4 (3.0)	42 (5.3)	
TAPP with general anesthesia	6 (4.5)	59 (7.4)	
Sufentanil, median (range), ug	30.0 (20.0-39.4)	30.0 (20.0-35.0)	0.432
Propofol, median (range), mg	698.3 (549.0-1001.7)	773.4 (619.1-943.9)	0.096
Dexmedetomidine, median (range), ug	43.3 (0.0-74.4)	51.4 (0.0-77.6)	0.482
Rocuronium, median (range), mg	120.0 (90.0-150.0)	95.0 (75.0-115.0)	<0.001
Intraoperative blood transfusion	9 (6.8)	61 (7.6)	0.860
Intraoperative fluid utilization, median (range), mL	2275.0 (2000.0-3000.0)	2000.0 (2000.0-2500.0)	0.644
Urinary Output, mL	400.0 (300.0-675.0)	400.0 (222.5-600.0)	0.925
Bleeding, mL	100.0 (85.0-200.0)	100.0 (50.0-100.0)	<0.05

CCI, charlson comorbidity index; NRS2002, nutritional risk screening 2002; Hct, hematocrit; Hgb, hemoglobin; Neut, neutrophils; Alb, albumin; POD, postoperative day; ΔALB, (preoperative Alb – nadir Alb within POD 2)/preoperative ALB × 100%.

### Univariate and multivariate analyses

**Figure 2:**
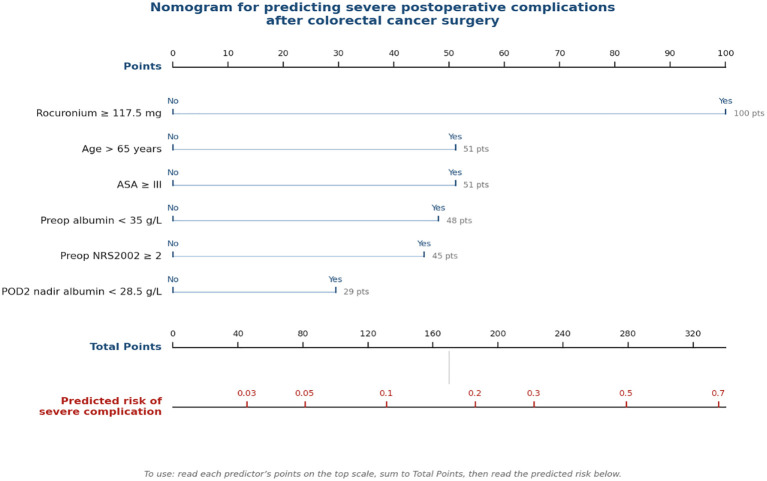
Nomogram predicting severe complications after colorectal cancer resection.

**Figure 3:**
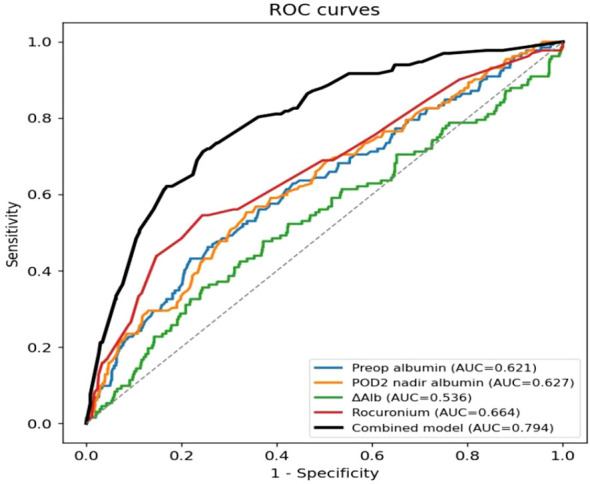
Receiver operating characteristic (ROC) curves for individual predictors (preoperative albumin, POD2 nadir albumin and rocuronium) and for DAlb, with the combined multifactorial model shown for comparison. DAlb discriminated near chance (AUC 0.536), whereas the combined model achieved the highest AUC (0.794). AUC = area under the curve.

Univariable analysis identified age >65 years, ASA ≥3, preoperative NRS2002 ≥2, preoperative haemoglobin, preoperative albumin <35 g/L, POD2 nadir albumin <28.5 g/L, laparoscopic approach and rocuronium ≥117.5 mg as factors associated with severe complications (all P<0.05). In the final multivariable model, age >65 years (OR 2.14, 95% CI 1.30–3.52, P = 0.003), ASA ≥3 (OR 2.14, 95% CI 1.36–3.37, P = 0.001), preoperative NRS2002 ≥2 (OR 1.97, 95% CI 1.08–3.60, P = 0.028), preoperative albumin <35 g/L (OR 2.05, 95% CI 1.29–3.25, P = 0.003), POD2 nadir albumin <28.5 g/L (OR 1.55, 95% CI 1.01–2.38, P = 0.044) and rocuronium ≥117.5 mg (OR 4.44, 95% CI 2.92–6.75, P<0.001) remained independently associated with severe complications (**Table 3**); ΔAlb was not retained (univariable P = 0.185). The model was visualised as a nomogram (**Figure 2**), with the scoring method in **Table 4**. Discrimination was good (apparent C-index 0.794; optimism-corrected 0.784 after 1000-sample bootstrapping; bootstrap calibration slope 0.954); calibration was satisfactory (Hosmer–Lemeshow P = 0.72; Brier score 0.102) and decision-curve analysis showed positive net benefit across threshold probabilities of approximately 5–50% (**Figure 3**). For example, a 70-year-old patient with ASA 3, preoperative NRS2002 <2, preoperative albumin 30 g/L, POD2 nadir albumin 35 g/L and intraoperative rocuronium 120 mg scores 250 points, corresponding to a predicted probability of approximately 0.4 for severe complications.

**Table 3:** Univariate and multivariate analyses of perioperative factors on the development of postoperative severe complications.

Characteristic	Univariable OR (95% CI)	P value	Multivariable OR (95% CI)	P value
Age >65 y	3.73 (2.49–5.60)	<0.001	2.14 (1.30–3.52)	0.003
ASA ≥III	3.18 (2.13–4.74)	<0.001	2.14 (1.36–3.37)	0.001
Preoperative NRS2002 ≥2	4.21 (2.48–7.15)	<0.001	1.97 (1.08–3.60)	0.028
Preoperative albumin <35 g/L	2.27 (1.53–3.37)	<0.001	2.05 (1.29–3.25)	0.003
POD2 nadir albumin <28.5 g/L	2.35 (1.62–3.41)	<0.001	1.55 (1.01–2.38)	0.044
Rocuronium ≥117.5 mg	3.72 (2.55–5.44)	<0.001	4.44 (2.92–6.75)	<0.001
Preoperative Hgb, g/L	0.99 (0.98–1.00)	0.027	—	—
Laparoscopic surgery	1.82 (1.10–3.02)	0.020	—	—

Final model uses clinically dichotomised predictors to match the nomogram. ΔAlb was non-significant (univariable OR 1.01, P = 0.185) and not retained; the continuous-variable model is reported as a sensitivity analysis (**Supplementary Table 1**). NRS2002, nutritional risk screening 2002; Hgb, haemoglobin; POD, postoperative day.

**Table 4:** Nomogram score.

	Values	Points		Values	Points
Age	>65	51	Preoperative Alb	<35	48
	≤65	0		≥35	0
ASA	≥3	51	Nadir Alb within POD 2	<28.5	29
	<3	0		≥28.5	0
Preoperative NRS2002	≥2	45	Rocuronium	≥117.5	100
	<2	0		<117.5	0
	Values		Probability		
Total Points	131		0.1		
	179		0.2		
	224		0.3		
	250		0.4		
	279		0.5		
	295		0.6		
	324		0.7		

### ROC curve analysis

**Figure 4:**
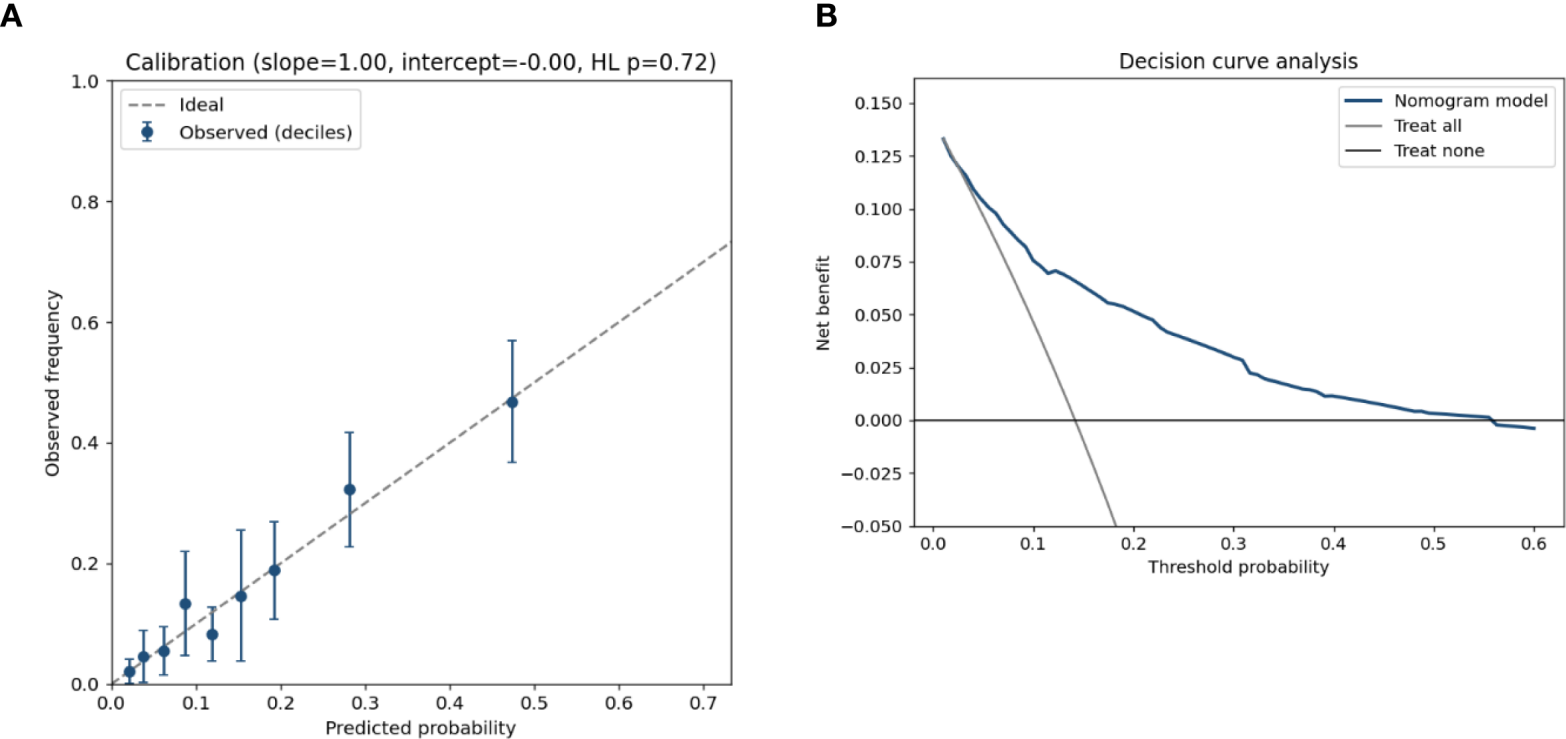
Internal validation of the nomogram. **(A)** Calibration plot of predicted versus observed risk of severe complications (deciles, with 95% CI; Hosmer–Lemeshow P = 0.72). **(B)** Decision-curve analysis showing net benefit of the model versus treat-all and treat-none strategies.

The predictive power of preoperative albumin, nadir albumin within POD 2, and rocuronium levels was evaluated through ROC curve analysis (**Figure 4**). The area under the ROC curve (AUC) for preoperative albumin was 0.621, with a sensitivity of 0.364 and specificity of 0.799 at a cutoff point of 35.0 g/L. For nadir albumin within POD 2, the AUC was 0.627, with a sensitivity of 0.558 and specificity of 0.669 at a cutoff point of 28.5 g/L. Rocuronium levels had the highest AUC of 0.664, with a sensitivity of 0.545 and specificity of 0.756 at a cutoff point of 117.5 mg. The Youden index values for preoperative albumin, nadir albumin, and rocuronium were 0.162, 0.207, and 0.301, respectively, indicating moderate predictive ability (**Table 5**). On formal comparison, ΔAlb discriminated no better than chance (AUC 0.536, 95% CI 0.479–0.593). By DeLong’s test, POD2 nadir albumin and rocuronium each significantly outperformed ΔAlb (both P<0.001), whereas the difference between preoperative albumin and ΔAlb did not reach significance (P = 0.074).

**Table 5:** Diagnostic performance of preoperative albumin, POD2 nadir albumin and rocuronium dose for predicting severe postoperative complications, with DeLong comparison against ΔAlb.

	Preoperative Alb	Nadir Alb within POD 2	Rocuronium
Cutoff point	35.0	28.5	117.5
AUC	0.621	0.627	0.664
True positives	48	71	72
False positives	161	265	195
True negatives	639	535	605
False negatives	84	61	60
Sensitivity	0.364	0.538	0.545
Specificity	0.799	0.669	0.756
Positive predictive value	0.230	0.211	0.270
Negative predictive value	0.884	0.898	0.910
Youden index	0.162	0.207	0.301
DeLong vs ΔAlb	0.074	<0.001	<0.001

### Model validation

Internal validation by 1000-sample bootstrapping yielded an optimism-corrected C-index of 0.784 (apparent 0.794; optimism 0.010) and a calibration slope of 0.954, indicating minimal overfitting. The calibration plot (**Figure 3A**) showed close agreement between the predicted and observed risk of severe complications across deciles of predicted risk, with no evidence of systematic miscalibration (Hosmer–Lemeshow P = 0.72; Brier score 0.102; calibration intercept ≈0 and slope ≈0.95). Decision-curve analysis (**Figure 3B**) demonstrated that, across threshold probabilities of approximately 5–50%, the nomogram provided greater net clinical benefit than the “treat-all” and “treat-none” strategies, supporting its potential value for risk stratification within this range.

### Subgroup analysis

**Figure 5:**
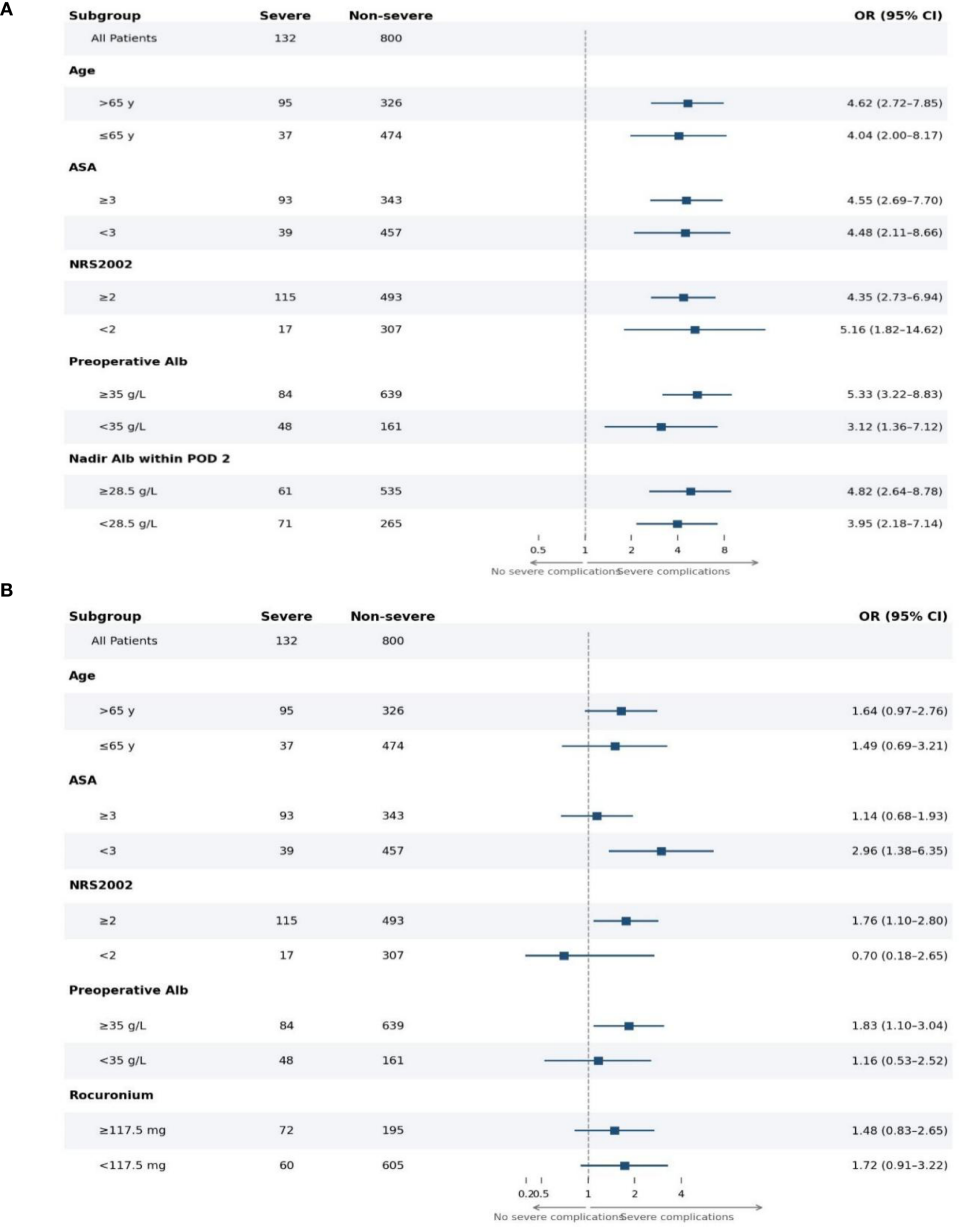
Subgroup analysis of severe complications. **(A)** Subgroup analysis of patients stratified by total intraoperative rocuronium dose (≥117.5 mg). **(B)** Subgroup analysis of patients stratified by nadir albumin levels ≤28.5 g/L within POD 2. OR, odds ratios; CI, 95% confidence intervals.

The odds ratio of intraoperative rocuronium ≥117.5 mg for severe complications was consistently elevated (approximately 4–5) across all subgroups (**Figure 5A**): age >65 years (OR 4.62, 95% CI 2.72–7.85), ASA ≥3 (OR 4.55, 2.69–7.70), NRS2002 ≥2 (OR 4.35, 2.73–6.94), preoperative albumin ≥35 g/L (OR 5.33, 3.22–8.83) and POD2 nadir albumin ≥28.5 g/L (OR 4.82, 2.64–8.78). The association remained significant even in the lower-risk strata (e.g. ASA <3, OR 4.48, 2.11–8.66; preoperative albumin <35 g/L, OR 3.12, 1.36–7.12), indicating that higher rocuronium dose is associated with severe complications across the whole cohort rather than only in selected vulnerable populations.

Stratified by POD2 nadir albumin <28.5 g/L (**Figure 5B**), low early postoperative albumin remained significantly associated with severe complications among patients with NRS2002 ≥2 (OR 1.76, 95% CI 1.10–2.80), preoperative albumin ≥35 g/L (OR 1.83, 1.10–3.04) and ASA <3 (OR 2.96, 1.38–6.35). The association was attenuated and non-significant in the ASA ≥3 (OR 1.14, 0.68–1.93) and age ≤65 years strata, suggesting that early postoperative hypoalbuminemia contributes prognostic information beyond baseline comorbidity and age. As this is a stratified retrospective analysis, these subgroup associations should be regarded as exploratory.

### Correlation of risk factors with outcome measures

**Figure 6:**
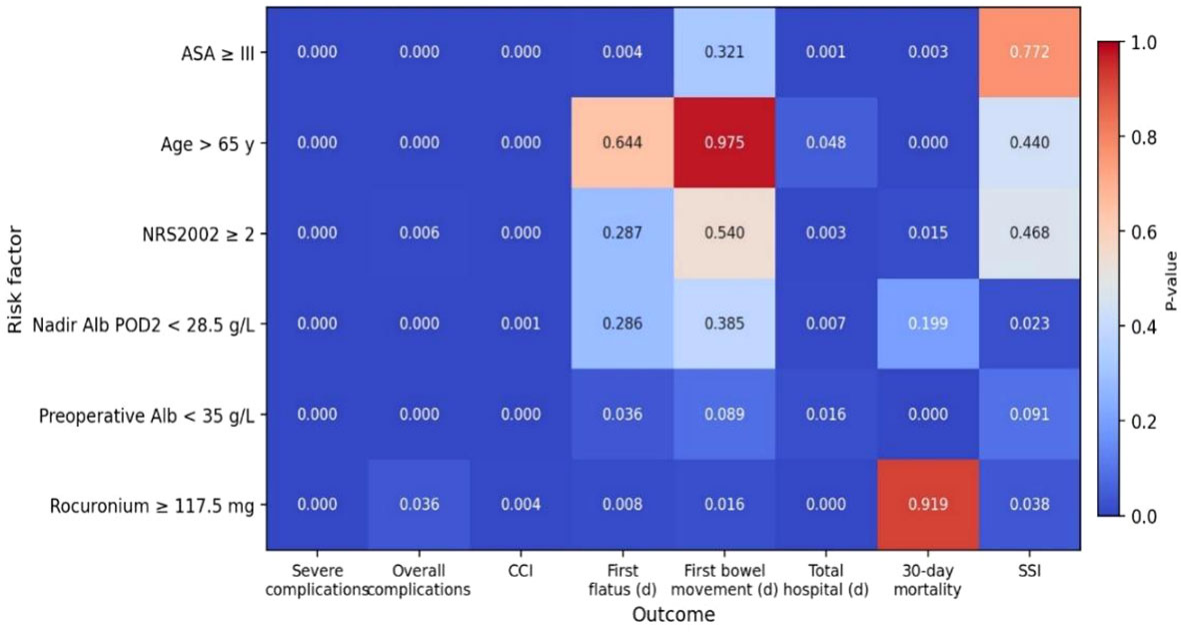
Heatmap of p-values for risk factors associated with surgical outcomes.

A heatmap was used to analyze the statistical correlations between various risk factors and outcome measures (**Figure 6**), including severe complications, overall complications, Clavien-Dindo classification, time to first flatus, time to first defecation, total hospital stay, 30-day mortality rate, and surgical site infections (SSI).

Notably, ASA ≥3 was significantly associated with nearly all adverse outcomes (p < 0.05), especially severe complications, overall complications, and prolonged hospitalization. NRS2002 ≥2, preoperative albumin <35 g/L, and nadir albumin within POD 2 <28.5 g/L were consistently strong predictors of complications and delayed recovery, with significant associations found across various outcomes, including time to first flatus and bowel movement, prolonged hospitalization, and 30-day mortality (p < 0.05 for each).

However, age >65 years demonstrated a more limited impact on specific outcomes, with non-significant associations for certain parameters such as first bowel movement (p = 0.975) and first flatus (p = 0.644), while retaining significant associations with overall complication rates (p < 0.001) and 30-day mortality (p = 0.048).

## Discussion

### Principal findings

This study evaluated the association between preoperative serum albumin levels, their postoperative dynamic changes, and the occurrence of severe and overall complications following colorectal cancer surgery. The results indicated that while preoperative and the lowest albumin levels on postoperative day 2 had some predictive value individually, integrating multiple preoperative and intraoperative risk factors significantly improved predictive accuracy. Specifically, key risk factors included age > 65 years, ASA score ≥ 3, NRS2002 score ≥ 2, preoperative albumin level ≤ 35.0 g/L, lowest albumin level on postoperative day 2 ≤ 28.5 g/L, and intraoperative rocuronium dosage ≥ 117.5 mg.

### Comparison with previous evidence

The results of this study indicate that perioperative albumin change (ΔAlb) alone has limited predictive value for postoperative complications, despite being proposed as a potentially useful biomarker in recent literature. While several studies have demonstrated significant associations between ΔAlb and complications following various types of abdominal surgery (12, 17–19), the evidence remains inconsistent. Mantziari et al. found that ΔAlb was not an independent risk factor in a prospective study of abdominal surgery patients (20), and Gaspar-Figueiredo et al. reported similar findings in pancreatic surgery (9). Although Hendifar et al. identified an association between ΔAlb and disease-free survival (21), its predictive ability for postoperative complications remained unclear. This inconsistency across studies, combined with the significant heterogeneity in reported ΔAlb thresholds, has limited its clinical applicability and prompted us to investigate the underlying reasons for these discrepant findings. This limited reliability likely reflects albumin’s long half-life (approximately 20 days), as early postoperative changes are driven largely by haemodilution, capillary leak, intraoperative fluid administration and transiently suppressed hepatic synthesis rather than by complication risk itself.

Serum albumin is a nonspecific marker influenced by nutrition, systemic inflammation, capillary leak, hepatic synthetic function, hydration status and overall disease burden rather than by nutrition alone (22, 23); it is therefore better regarded as a surrogate of frailty, inflammation and physiological reserve. Low albumin may be accompanied by immunosuppression, delayed wound healing and an increased risk of infection (24). A decline in the lowest albumin level on postoperative day 2 may result from inflammatory responses triggered by surgical trauma and suppressed hepatic synthesis (25). Moreover, vasodilator use and fluid management during the perioperative period may lower colloid osmotic pressure (26), contributing to oedema and wound exudation that may, in turn, be associated with infection, bleeding and anastomotic leakage (27, 28). These results indicate an association, rather than a causal relationship, between perioperative albumin reduction and postoperative complications.

Beyond albumin in isolation, combined inflammation–nutrition indices such as the C-reactive protein-to-albumin ratio (CAR) offer an elegant way to capture both the systemic inflammatory response (CRP) and physiological–nutritional reserve (albumin) within a single, easily obtained measure. In a recent and directly relevant study, Evirgen and Çetin demonstrated that the postoperative day-7 CAR was strongly associated with surgical complications after elective colorectal cancer surgery: mean CAR was more than twice as high in patients who developed complications (5.07 vs 2.31), and a CAR ≥3.2 identified a markedly higher complication rate (74.4% vs 14.0%) (29). Their work elegantly illustrates the prognostic power of integrating inflammation and nutritional status, and provides an important reference point for the present study. Our findings are highly complementary to theirs. Whereas their analysis highlights the value of the combined CAR signal as it matures in the later postoperative period—around day 7, by which time many patients managed with enhanced-recovery pathways have already been discharged—our model focuses on preoperative and early postoperative (POD2) absolute albumin thresholds together with intraoperative factors, enabling risk stratification at an earlier time point. In keeping with their observation that the inflammatory–nutritional signal strengthens after surgery, we found that absolute preoperative albumin already carried independent prognostic information in our cohort, suggesting that albumin is informative across the entire perioperative continuum. Building on this body of work, we additionally incorporated multivariable modelling, an internally validated nomogram and a formal AUC comparison (DeLong’s test) to estimate the adjusted contribution of each marker. As C-reactive protein was not routinely available in our retrospective dataset—already acknowledged as a limitation—we were unable to compute CAR directly; we therefore regard the integration of serial CRP and CAR, as advocated by Evirgen and Çetin, with absolute albumin thresholds and intraoperative variables as a particularly promising direction for future prospective studies.

The results of this study indicate a significant correlation between increased intraoperative rocuronium dosage and the incidence of postoperative complications. Although anesthetic dosage is not typically considered an independent predictor of postoperative risk, the administration of high-dose rocuronium may reflect greater surgical complexity and prolonged operative time. Notably, operative time did not differ significantly between groups with and without severe complications (median 220 vs. 219 min, P = 0.630), suggesting that the observed risk associated with higher rocuronium dosage should be interpreted as being associated with, rather than causally related to, severe complications. Higher cumulative dose may act as a surrogate for surgical complexity, operative duration and difficulty, patient body weight, anaesthesiologist preference, and neuromuscular monitoring and reversal practices, and residual confounding cannot be excluded in this retrospective design. Additionally, it may contribute to delayed muscle function recovery and respiratory impairment, leading to muscle weakness, dyspnea, and prolonged recovery (30–32). Thevathasan et al. found a dose-dependent relationship between neuromuscular blocker dosage and an increased risk of hospital readmission within 30 days, prolonged hospitalization, and higher medical costs (33), supporting the hypothesis that high-dose rocuronium may lead to complications and long-term adverse outcomes. In morbidly obese patients undergoing bariatric surgery, deep neuromuscular blockade has been found to improve laparoscopic surgical conditions and reduce postoperative pain and facilitate low-pressure pneumoperitoneum compared with moderate blockade (34), possibly due to increased intra-abdominal pressure and thickened abdominal wall fat, necessitating greater muscle relaxation. Currently, standardized guidelines for neuromuscular blocker administration remain incomplete, with no unified dosage control framework beyond the initial intubation dose (35). Optimizing neuromuscular blockade use is particularly crucial in complex surgeries. When high-dose administration is unavoidable, dynamic adjustment of muscle relaxant dosage based on train-of-four (TOF) monitoring during major abdominal surgeries, followed by the use of neuromuscular reversal agents at the end of surgery, is recommended to mitigate residual neuromuscular blockade and associated postoperative complications (36, 37).

### What this study adds

However, rather than dismissing albumin as a predictive marker, our study refines its clinical application by demonstrating that absolute albumin levels provide more clinically meaningful information than relative changes. Specifically, our data show that preoperative albumin <35 g/L and POD2 nadir albumin <28.5 g/L are significant independent predictors of complications, whereas ΔAlb is not. This distinction is clinically relevant because absolute thresholds capture chronic nutritional and physiological-reserve deficits (preoperative albumin <35 g/L) and inadequate early postoperative status (POD2 nadir albumin <28.5 g/L) that, when combined with age >65 years, ASA ≥3, NRS2002 ≥2 and rocuronium ≥117.5 mg, yield substantially better discrimination than any single predictor. Therefore, our findings suggest that previous studies focusing solely on ΔAlb may have overlooked the more clinically relevant approach of integrating absolute albumin levels with other risk factors to create comprehensive prediction models.

Subgroup analysis revealed heterogeneity in risk across populations: among patients receiving rocuronium ≥117.5 mg, those with NRS2002 ≥2 or preoperative albumin <35 g/L were at particularly high risk, suggesting that poorer nutritional status lowers tolerance to higher drug doses; a similar interaction was observed between NRS2002 ≥2 and POD2 nadir albumin <28.5 g/L. The relative independence of early postoperative hypoalbuminemia from age and ASA further supports its role as a key risk marker and the value of individualised perioperative management in high-risk patients.

### Limitations

However, this study also has some limitations. First, it is a retrospective analysis, which inevitably means that potential confounding factors could not be entirely excluded. Additionally, because this is a single-center study, the generalizability of the results may be limited. Future large-scale multi-center studies are needed to validate these findings. Third, complications following colorectal surgery are multifactorial, influenced by patient, tumor, surgical, and perioperative factors. While our model incorporated multiple risk factors and achieved good discriminative ability (C-index 79.44%), unmeasured confounders such as surgeon experience, specific anastomotic techniques, and microbiome status could not be accounted for. Fourth, albumin levels are influenced by multiple factors beyond nutrition; we minimized confounding by excluding patients with severe organ dysfunction and exogenous albumin use, but residual confounding remains possible. Fifth, quantitative train-of-four (TOF) monitoring data were not systematically recorded in this retrospective cohort. Therefore, we cannot definitively exclude the possibility that higher rocuronium dosage was associated with subtle residual neuromuscular blockade at the time of extubation. This finding should be interpreted with caution and warrants prospective validation with standardized TOF-guided neuromuscular management. Other potentially relevant variables—including emergency versus elective status, neoadjuvant chemoradiotherapy, open conversion, C-reactive protein, perioperative fluid balance, and liver and renal function—were not available in this dataset. In addition, the continuous albumin and rocuronium variables were dichotomised at data-derived ROC cut-offs, which can cause information loss and optimism; although bootstrap correction indicated limited overfitting (optimism 0.010), the model was validated internally but not in an external cohort. Finally, the individual predictors showed only poor-to-modest discrimination (AUC 0.62–0.66) with low positive predictive value (approximately 13–16%); the nomogram is therefore intended for risk stratification and hypothesis generation rather than standalone clinical decision-making, pending external validation.

### Implications and future research

These findings carry several clinical implications. Because the nomogram relies on six routinely available perioperative variables, it could help flag patients at higher risk of severe complications early after colorectal cancer surgery and prompt closer postoperative surveillance, optimised perioperative nutritional and fluid management, and more judicious intraoperative neuromuscular blockade with quantitative train-of-four monitoring. As the model was derived and internally validated in a single-centre retrospective cohort and relies on data-derived cut-offs, prospective multicentre studies that incorporate standardised neuromuscular monitoring, inflammatory markers and additional surgical confounders are needed to externally validate and refine it before routine clinical adoption.

## Conclusion

This study indicates that preoperative albumin <35 g/L and preoperative NRS2002 ≥2 are significant risk factors for severe postoperative complications after colorectal cancer surgery. A multifactorial model combining POD2 nadir albumin <28.5 g/L with age >65 years, ASA ≥3, preoperative NRS2002 ≥2 and intraoperative rocuronium ≥117.5 mg improves risk prediction (optimism-corrected C-index 0.78). Because rocuronium dose may also reflect surgical complexity and perioperative management, and the model was internally but not externally validated, these findings should be interpreted as associations requiring prospective, multicentre validation before clinical use.

## Data Availability

The raw data supporting the conclusions of this article will be made available by the authors, without undue reservation.
