# Supplementary material for: Preoperative and early postoperative albumin thresholds, rather than ΔAlb, predict severe complications after colorectal cancer surgery: a multifactorial model and nomogram
Source: Front Oncol. 2026 Jul 13;16:1864078. doi: 10.3389/fonc.2026.1864078 (PMC13402124; doi:10.3389/fonc.2026.1864078)
Supplement: Supplementary file 1 [file Table1.docx]

**Supplementary Table S1.** Multivariable logistic regression using continuous albumin and rocuronium predictors (sensitivity analysis).

| **Variable** | **OR** | **95% CI** | **P value** |
| --- | --- | --- | --- |
| Age >65 y | 1.84 | 1.13–3.02 | 0.015 |
| ASA ≥III | 2.33 | 1.48–3.66 | <0.001 |
| Preoperative NRS2002 ≥2 | 2.06 | 1.13–3.76 | 0.019 |
| Preoperative albumin (per 1 g/L increase) | 0.948 | 0.898–1.001 | 0.054 |
| POD2 nadir albumin (per 1 g/L increase) | 0.942 | 0.893–0.994 | 0.030 |
| Rocuronium (per 1 mg increase) | 1.016 | 1.011–1.021 | <0.001 |

This continuous-variable model (apparent AUC 0.787) is concordant with the primary categorical model (Table 3; apparent AUC 0.794): lower albumin and higher rocuronium dose were associated with increased risk in both. The categorical model was retained as the primary analysis to match the nomogram and improve clinical usability. OR = odds ratio; CI = confidence interval.
